# Morphological configuration of the cranial base among children aged 8 to 12 years

**DOI:** 10.1186/s13104-016-2115-2

**Published:** 2016-06-14

**Authors:** Lina Cossio, Jorge López, Zulma Vanessa Rueda, Paola Botero-Mariaca

**Affiliations:** Universidad Cooperativa de Colombia, Carrera 47 # 37 South 18, Medellín, Antioquia Colombia

**Keywords:** Cranial base, Growth, Length, Mean values

## Abstract

**Background:**

Cranial base is used as reference structure to determine the skeletal type in cephalometric analysis. The purpose was to assess the cranial base length on lateral cephalic radiographs of children between 8 and 12 and compare these measurements with baseline studies in order to evaluate the relationship between the length and the cranial base angle, articular angle, gonial angle and skeletal type.

**Methods:**

A Cross-sectional study in 149 children aged 8–12 years, originally from Aburrá Valley, who had lateral cephalic radiographs and consented to participate in this study. The variables studied included: age, sex, sella–nasion, sella–nasion–articular, sella–nasion–basion, articular–gonion–menton, gonion–menton, sella–nasion–point B, sella–nasion–point A y point A-nasion–point B. These variables were digitally measured through i-dixel 2 digital software. One-way ANOVA was used to determine mean values and mean value differences. The values obtained were compared with previous studies. A p value <0.05 was considered significant.

**Results:**

Cranial base lengths are smaller in each age and sex group, with differences exceeding 10 mm for measurement, compared both with the study by Riolo (Michigan) and the study carried out in Damasco (Antioquia). No relation was found between the skeletal type and the anterior cranial base length, the sella angle and the cranial base angle. Also, no relation was found between the gonial angle and sella angle or the cranial base angle.

**Conclusion:**

The cranial base varies from one population to another. Accordingly, compared to other studies it is shorter for the assessed sample.

## Background

The anterior cranial base (sella–nasion) is an important component of the craniofacial structure because it influences both its dimension and growth orientation. It also serves as a reference to determine the size of both the maxilla and the mandible in lateral cephalic radiographs. Since it is considered stable, this structure is the basis for skeletal diagnose. Its linear size as well as the angle formed with the posterior cranial base (sella–basion) has been classified for certain populations as mean values by age for each sex [1]. Furthermore, a relation either positive or negative between the length and angulation of the cranial base and both the sagittal skeletal type and vertical growth has been reported by different studies [1].

**Fig. 1:**
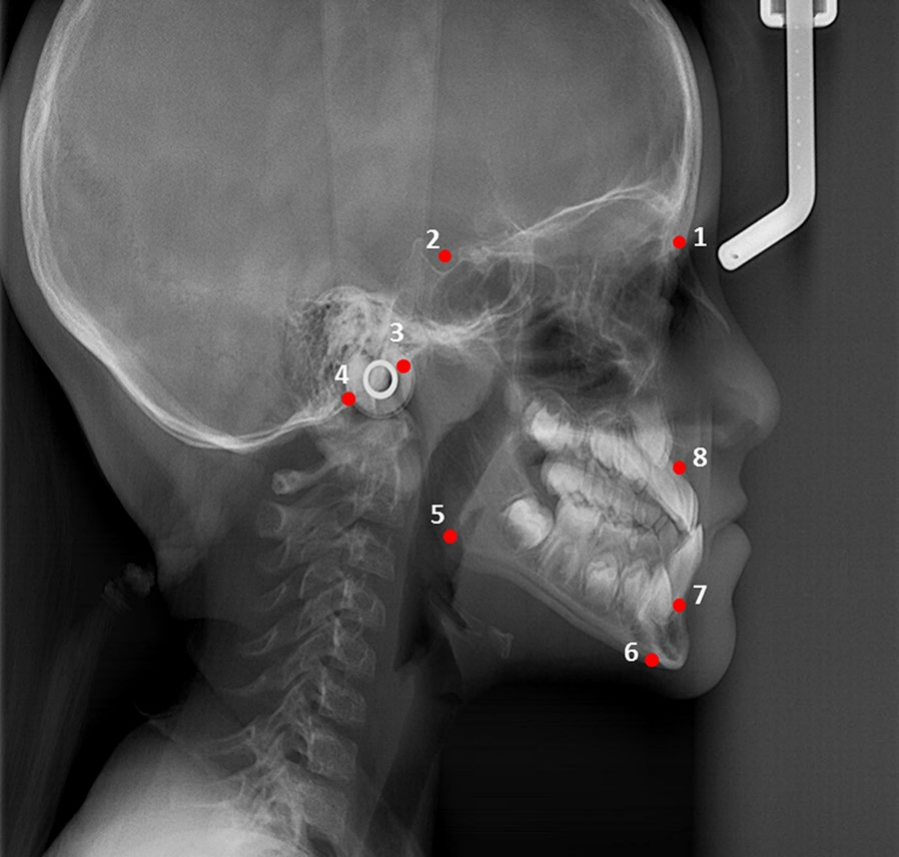
Location of reference points for lateral cephalic radiograph obtained for this study. *1* Nasion (N): junction of nasal and frontal bones. *2* Sella (S): midpoint of sella turcica. *3* Articular (Ar): point of intersection of the basilar apophysis of the occipital bone and the posterior border of the condyle. *4* Basion (Ba): most anterior inferior point of the anterior border of the occipital hole. *5* Gonion (Go): determined at the bisection of the angle formed by the posterior surface of the mandibular ramus and the mandibular body. *6* Menton (Me): lower most point of the mandibular symphysis curve. *7* B point (B): deepest point on anterior profile of mandibular symphysis. *8* A point (A): deepest point on anterior profile of superior maxilla

**Fig. 2:**
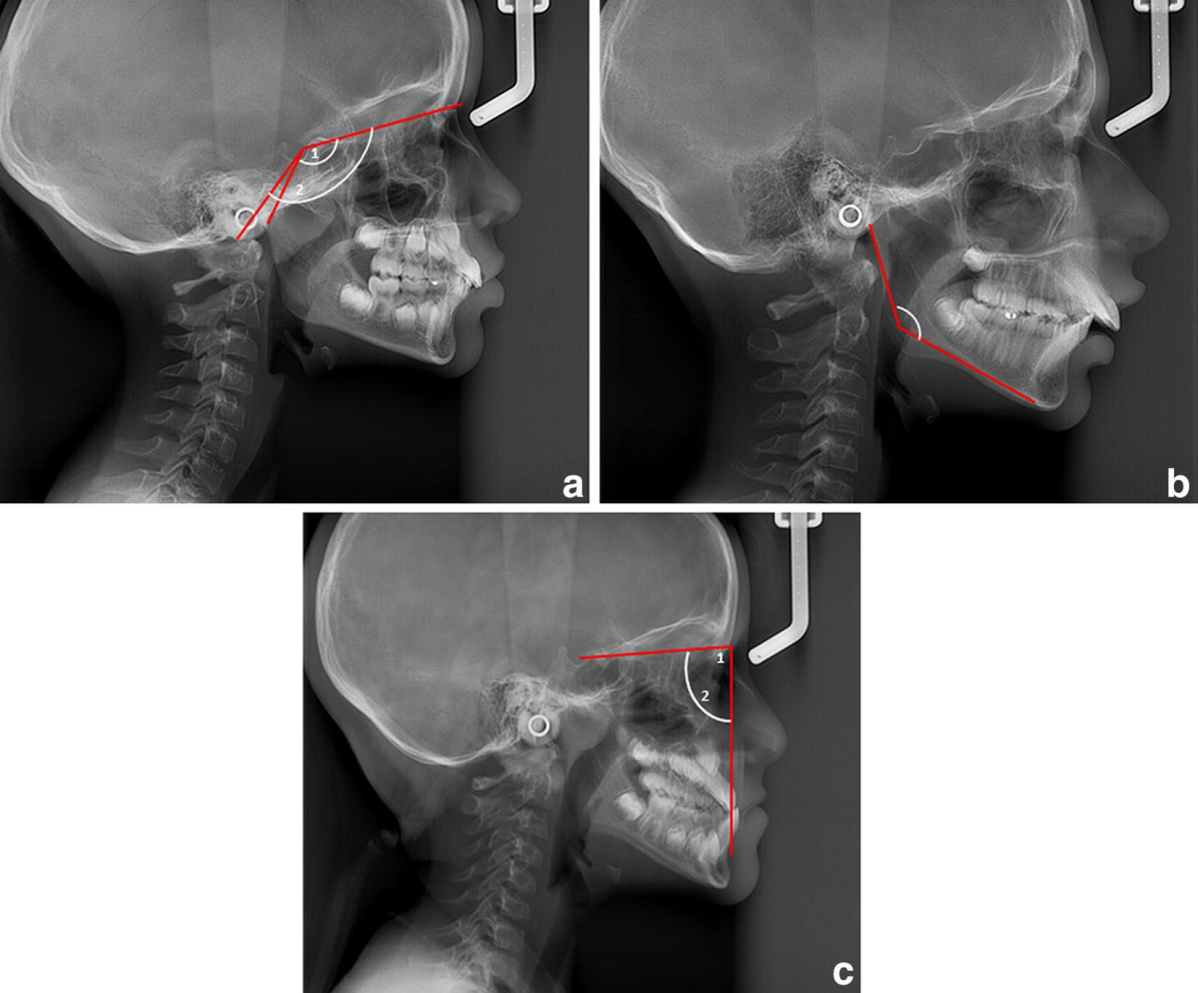
**a** Type I patient: *1* Measurement S-N-Ba angle. *2* Measurement S-N-Ar angle. **b** Type II patient: Measurement Ar-Go-Me angle. **c** Type III patient: *1* Measurement SNA angle. *2* Measurement SNB angle

In a cephalometric analysis three types of skeletal anteroposterior relationships can be diagnosed, type I relationship when the maxilla and mandible have normal anteroposterior position (Average ANB), type II relationship when the mandible is positioned distally to the maxilla (larger ANB than average) and type III when mandible is mesially positioned to the maxilla (decreased ANB than average) (Figs. 1, 2).

The anterior cranial base may have different mean values for sizes according to the population where the study is carried out. For example, Bolton in Ohio and Riolo in Michigan found significant differences when comparing the length of the sella-nasion plane [2, 3]. Several studies have been carried out in Colombia, but these results have not yet been taken into account to implement applicable mean values for our population. The reasons for this are: sample size, type of methodology used, as well as variation among X-ray equipment. Some of these studies include Zagarra and Villegas in Bogotá [4], Cárdenas in Heliconia (Antioquia) [5], Palacino in Medellín [6] and Botero et al. in Damasco, Antioquia [7].

Cranial base length and flexure influences jaw relationship, glenoid fossa sagittal position among others. A decreased basicranial angulation has been related with type III mandibular position. Steeper posterior cranial base, more inferiorly positioned sphenoidale and more anteriorly positioned basion are major characteristics of type III [8–11]. In cephalometric analysis the determination of skeletal relationships between maxilla and mandible are established using cranial base as a reference structure. Cranial base growth and development can have a genetic influence and therefore have a specific configuration depending upon the genetic ancestry. Cranial base length in individuals form Aburra Valley is different from previous studies, given the variability between growth patterns in different populations. The aim of this study was to assess the cranial base length on lateral cephalic radiographs of children aged between 8–12 in order to compare them with other baseline studies and evaluate the relation between the length and the cranial base angle, the articular angle, the gonial angle and the skeletal type based on the ANB angle.

## Methods

### Type of study

Cross-sectional.

### Population

Children aged 8–12 who were scheduled for lateral cephalic radiographs. The sample collection was carried out in a radiology center of the city. The sample was collected and measured from February 29, 2012, to April 25, 2013. While the researchers of the present study did not prescribe the radiographs, written informed consent was obtained from all parents or legal guardians to assess their radiographs. This study is in compliance with the ethical requirements provided by Resolution 8430 of 1993, issued by the Ministry of Health of Colombia, and was approved by the Ethics Committee of the Universidad Cooperativa de Colombia, Medellín.

### Sample size

It was calculated based on the mean values and standard deviations for the sella-nasion obtained for every age and sex range, from the studies by Riolo in Michigan [3] and Botero et al. in Damasco, Antioquia [7]. With a confidence level of 95 %, an estimated loss of information of 20 % and accuracy level of 2 %, a sample of 148 patients was obtained, according to the data of the study carried out by Riolo, and 58 patients according to the study by Botero et al. The higher size was taken for this study. Estimation of sample size was done with the EPIDAT 3.1 software, which yielded a sample size of 149 radiographs.

### Inclusion criteria

Boys and girls aged 8-12 who attended the radiology center of the dental school at Universidad Cooperativa de Colombia, in order to get a lateral cephalic radiograph taken; who agreed to take art in the study and were born in the Aburrá Valley.

### Exclusion criteria

Patients with syndromes involving craniofacial structures, black race patients and lateral cephalic radiographs with structures that rendered location of cephalometric points impossible.

### Procedures

The lateral cephalic radiographs were obtained with MORITA Veraviewepocs 2D, with an exposure time of approximately 4.9 s and a constant magnification of 10.9 %. For measurements, i-Dixel software was used. A bone filter was applied for improved contrast and more accurate location of structures.

### Variables

The following were the cephalometric measurements assessed: Sella–nasion (S-N), Sella–nasion–basion (S-N-Ba), Sella–nasion–articular (S-N-Ar), Sella–nasion–A point (SNA), Sella–nasion–B point (SNB), Articular–gonion–menton (Ar-Go-Me) vertical rotation and ANB angle. Outcomes: type I when AB is average, type II when ANB is larger, type III when ANB is decreased. Neutral rotation when Ar-Go-Me is coincident with average, Ar-Go-Me larger than the average shows vertical rotation and Ar-Go-Me reduced is a horizontal rotation.

### Statistical analysis

Only one researcher performed the measurements in order to avoid variations from one person to another. Prior to the start of the study, radiograph readings were standardized between one of the expert researchers and the researcher responsible for the measurements. Interobserver concordance was assessed for each of the cephalometric measurements above, by using the intraclass correlation coefficient with mixed effects.

All cephalometric variables showed normal distribution. Therefore, the results are reported as mean values and standard deviations, stratified by age and gender. Subsequently, mean differences were tested through Student t test, in order to compare the values obtained in the present study with those obtained by Riolo in Michigan [3] and Botero et al. [7] in Damasco, Antioquia. Finally, one-way ANOVA tests were used to compare mean values of the cranial base length (S-N), the articular angle (Ar-Go-Me), and the S-Na-Ba angle with the ANB skeletal type (I, II, III) and the gonial angle (neutral, horizontal and vertical). The significance level was 0.05. The database was processed in Excel^®^, while the data analysis was performed through SPSS 20.0.

## Results

The interobserver concordance value for cephalometric measurements for standardization was higher than 0.8 for each of the variables (S-N: 0.99, S-N-Ar: 0.96, S-N-Ba, 0.92, SNA: 0.95, SNB: 0.91, Go-Me: 0.82, y Ar-Go-Me: 0.87), indicating almost perfect concordance. Consequently, the measurements performed were reliable.

A total of 149 children were included of those who went to the radiology center and met the inclusion criteria. Gender distribution was similar for each age range (Table 1).

**Table 1:** Children included in the study by gender and age

Age	Male	Female	Total
8	16	16	32
9	20	20	40
10	18	22	40
11	14	10	24
12	6	7	13
Total	74	75	149

The mean value for the cranial base was significantly lower across all age and gender groups of the present study, compared to the research by Riolo [3]. Also, the sella–nasion angle was wider among 11 year-old boys, while the sella–nasion–articular angle was wider among 10 and 11 year-old boys (Table 2).

**Table 2:** Comparison of sella–nasion, sella–nasion–basion and sella–nasion–articular measurements of this study and the research by Riolo [3]

Measurements	Current study					Riolo				p value	
	Age (years)	Female		Male		Female		Male			
		Mean value	SD	Mean value	SD	Mean value	SD	Mean value	SD	Female	Male
Sella nasion	8	60.23	2.8	60.91	2.11	72.3	2.9	75.2	3.0	<0.001	<0.001
	9	60.57	2.7	62.48	2.0	72.6	2.7	75.9	3.3	<0.001	<0.001
	10	62.22	2.2	64.22	2.0	73.9	2.8	76.8	3.2	<0.001	<0.001
	11	62.38	3.7	62.11	2.4	74.3	3.0	78.2	2.9	<0.001	<0.001
	12	63.54	3.0	64.27	1.4	74.9	3.0	78.3	3.3	<0.001	<0.001
Sella nasion/basion	8	133.36	7.57	130.46	5.15	130.0	4.8	129.0	4.8	0.117	0.311
	9	130.96	4.9	129.25	5.21	129.8	4.6	129.6	4.6	0.39	0.785
	10	131.19	4.45	130.99	4.13	129.7	4.5	129.2	4.7	0.22	0.162
	11	128.22	3.76	132.94	4.92	129.9	4.8	128.9	4.8	0.32	0.009
	12	130.07	6.2	132.20	4.76	130.4	5.2	129.3	4.8	0.88	0.171
Sella nasion/articular	8	124.52	4.26	124.28	4.62	123°	5	123°	5	0.31	0.296
	9	125.18	5.43	124.13	7.0	123°	5	123°	5	0.14	0.457
	10	125.16	5.7	126.03	5.2	123°	5	123°	5	0.13	0.035
	11	122.9	4.2	128.72	4.0	123°	5	123°	5	0.95	0.0003
	12	124.40	6.6	126.04	4.11	123°	5	123°	5	0.54	0.162

When comparing the results with the study by Botero et al. [7], all measurements for anterior cranial base obtained in the present study were observed to be significantly lower than those reported by the aforementioned authors (Table 3).

**Table 3:** Sella–nasion comparison: between this study and the study by Botero et al. [7]

Measurements	Current study					Botero et al.				p value	
	Age (years)	Female		Male		Female		Male			
		Mean value	SD	Mean value	SD	Mean value	SD	Mean value	SD	Female	Male
Sella-nasion	8	60.23	2.8	60.91	2.11	67.5	3.96	68.33	3.80	<0.001	0.0003
	10	62.22	2.2	64.22	2.0	69	3.25	69.65	3.88	<0.001	0.0003
	12	63.54	3.0	64.27	1.4	69.5	5.44	70.75	2.81	0.04	0.0002

Nine children out of 149 were classified as skeletal type I, 66.7 % of these exhibited a normal skull base angle, 21.1 % exhibited an increased angle and only 12.2 % exhibited a decreased angle. Twenty-five children were classified as skeletal type II, 44 % of these exhibited a normal skull base angle, 48 % exhibited an increased angle and only 2 % decreased angle. Finally, 34 children were classified as skeletal type III, 55 % of these exhibited a normal skull base angle, 38.2 % exhibited an increased angle and 5.9 % exhibited a decreased angle.

**Fig. 3:**
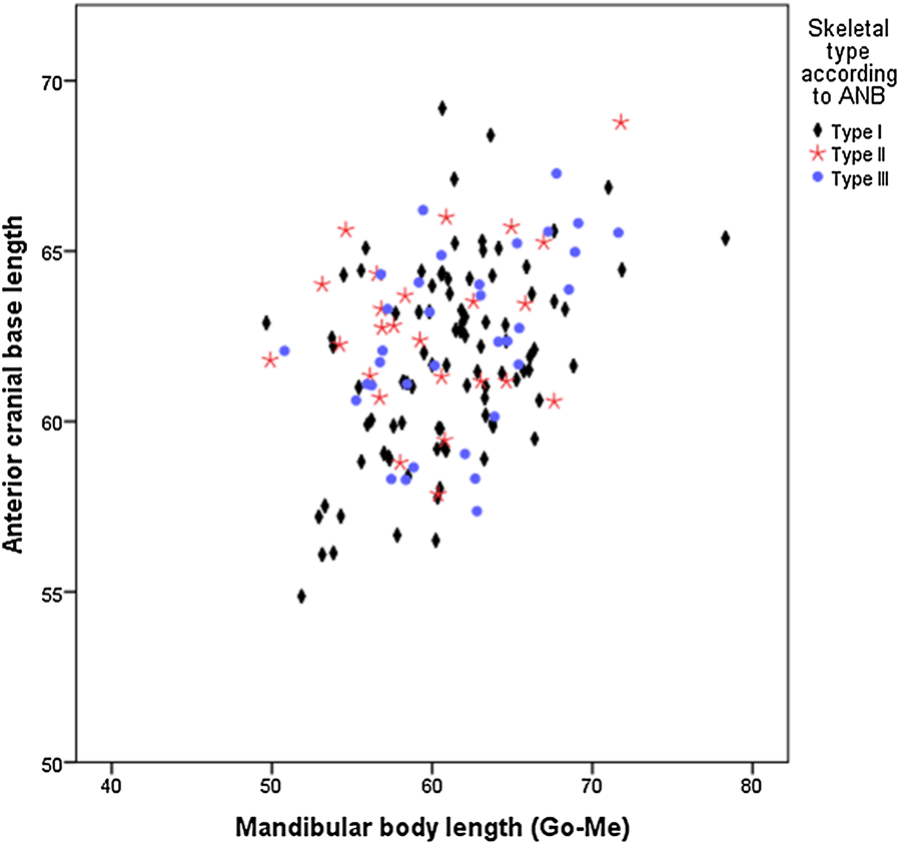
Relation between the anterior cranial base length and the mandibular body length classified as per skeletal type

When comparing mean values for the cranial base with the skeletal type, a relation was found between an increased length of the skull base and an increased length of the mandibular body for type I subjects (r = 0.435; p ≤ 0.001) and type III subjects (r = 0.438; p = 0.010). As for type II subjects, no correlation was observed between an increased skull base and the mandibular body (r = 0.258; p = 0.213) (Fig. 3 and Table 4).

**Table 4:** Comparison of skeletal type, articular angle and base skull angle with other cephalometric measurements assessed

	Mean differences	CI 95 % for mean differences		p value
		Lower limit	Upper limit	
Skeletal type according to ANB with skull base angle				
Type I vs type II	0.054	−2.82	2.93	1.0
Type I vs type III	−0.328	−2.88	2.23	1.0
Type II vs type III	−0.382	−3.73	2.96	1.0
Skeletal type according to ANB with anterior cranial base length				
Type I vs type II	−0.97	−2.46	0.52	0.349
Type I vs type III	−0.68	−2.00	0.65	0.652
Type II vs type III	0.29	−1.44	2.02	1.0
Articular angle with mandibular body length (Go-Me)				
Normal vs increased	−1.85	−3.98	0.28	0.112
Normal vs decreased	0.14	−3.09	3.37	1.000
Increased vs decreased	1.99	−1.47	5.45	0.497
Articular angle with gonial angle (Ar-Go-Me)				
Normal vs increased	1.26	−1.16	3.69	0.625
Normal vs decreased	0.16	−3.51	3.83	1.0
Increased vs decreased	−1.10	−5.04	2.83	1.0
Base angle with mandibular body length (Go-Me)				
Normal vs decreased	−0.41	−3.58	2.75	1.0
Normal vs increased	−0.29	−2.65	2.07	1.0
Increased vs decreased	0.12	−3.46	3.70	1.0
Skull base angle with gonial angle (Ar-Go-Me)				
Normal vs increased	3.59	0.10	7.09	0.04
Normal vs decreased	0.65	−1.95	3.26	1.00
Increased vs decreased	−2.94	−6.89	1.01	0.22

**Fig. 4:**
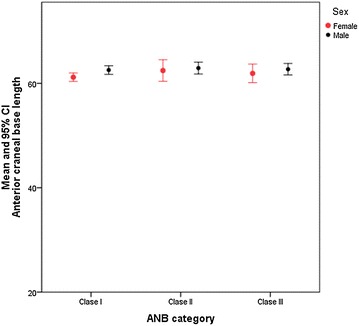
Relation between the anterior cranial base length and the skeletal type

No differences were found between the cranial base length and the skeletal type I, II and III, and stratified by men and women (Fig. 4 and Table 5).

**Table 5:** Cephalometric measurements based on the skeletal type, articular angle and skull base angle

	Skull base angle (sella/basion/nasion)		Anterior cranial base length	
	Mean value	SD	Mean value	SD
ANB skeletal type				
Type I	130.92	5.61	61.75	2.82
Type II	130.87	4.91	62.72	2.47
Type III	131.25	4.42	62.43	2.59

	Mandibular body length (Go-Me)		Gonial angle (Ar-Go-Me)	
	Mean value	SD	Mean value	SD
Articular angle				
Normal	60.47	4.65	128.86	5.75
Increased	62.32	5.00	127.59	4.47
Decreased	60.32	4.89	128.70	6.09
Normal skull base angle (sella/basion/nasion)				
Normal	60.89	4.46	129.00	5.41
Increased	61.18	5.83	128.35	4.32
Decreased	61.30	5.03	125.41	6.80

No differences were observed between the skeletal type and the articular angle and the skull base angle. Also, no differences were found between the articular angle and the skull base angle with the gonial angle or the mandibular body length (Table 4).

Type II patients exhibited increased articular angle, which indicates that the position of the glenoid cavity is more posteriorly located and, consequently, so it is the mandibular position; which can be related to the skeletal pattern exhibited.

## Discussion

The anterior cranial base is an important structure for cephalometric diagnosis due to its being considered a stable reference to determine the relationship between the maxilla and the skull (SNA, SNB, SNAr, SNBa), both in the sagittal and vertical dimensions. It also allows establishing positional and rotational diagnosis. Locating the points comprising the posterior cranial base on a lateral cephalic radiography has been controversial. Some authors, including Dhopatkar, Dibbets, Varjanne and Kerr, claim this structure starts at basion point [1, 12–14], while Bjork [8] and Anderson [13] use the articular point, which they consider easier to locate. However, the basion point is more appropriate given its anatomical location, its proximity to the skull base and also because it is an anatomical point rather than a cephalometric point [12, 13, 15]. In the present study both points were used to determine the cranial base angle and when compared no significant differences were found between them or in relation to the values yielded by Riolo’s study [3].

The cranial base length can be influenced by race [2, 3, 7]. Most studies determining average lengths based on age have been performed on Caucasian populations [2, 3]. It is, thus, important to determine mean cranial base lengths for our population in order to achieve a more accurate sagittal diagnosis of skeletal malocclusion. At the same time, there may be variations in the mean values within a population [2, 3]. This was demonstrated in the studies by Riolo [3] and Bolton [2], which were carried out in the USA. These authors conclude that there are significant differences (going up to 8 mm) for the sella-nasion measurement, with Bolton measurement being higher. Similar results have been reported in Colombia in the study by Botero, carried out in Damasco, Antioquia [7]. This study found significant differences with the study by Riolo [3], in which values were lower (up to 8 mm) for most dimensions; values being higher for men than for women.

The present study, carried out only with subjects born in Aburrá Valley, found that the cranial base length was significantly higher compared to the study by Riolo [3] (10 mm) and Botero (7 mm) [7].

These reported differences, including the one found with the Colombian sample, are attributed both to race variability of each population group and to the genetic admixture it contains. According to some population genetics studies, the population of the metropolitan area of Aburrá Valley has a European ancestral component of 70 %, an Amerindian component of 30 % and an African component of 10 %, with a deviation of ±10 for each percentage [14–16]. Accepting only individuals from the same geographical area can provide certainty of working with individuals who show similar environmental influences and equal genetic ancestry. Having a cranial base size above the average makes the analysis inaccurately result in a maxillary and mandibular protrusion relative to the skull. Furthermore, interpreting cephalometric analysis based upon mean values taken from different population can induced a skeletal misdiagnosis; for example individuals with type I characteristics can be diagnosed as type II when the cranial base length is compared with other population standards given the fact that is bigger than the average [10].

On the other hand, when relating the cranial base length with the mandibular length and the skeletal type (ANB), the results found were similar to those reported by Bjork [12, 17] and Kasay [18], who show that there is a relation between mandibular prognathism and the cranial base length. This differs from the results obtained by Wilhelm et al., who found no significant differences between the different skeletal types and the cranial base measurements [19], but is similar with other studies [8–11] who showed that type III patients have more acute basicranium angle and shorter cranial length. Besides type I patients exhibited a shorter cranial base than the type II and III patients; with no difference between these latter groups. This finding is in disagreement with what other studies reported where type III patients exhibited a reduced cranial base length [19]. Cranial base could influence mandibular pragmatism because it determines the antero-posterior location of the condyle related to facial profile [8].

While cranial base flexure can be associated to a specific facial pattern, its role as an etiological factor of sagittal discrepancies is limited and therefore controversial [16]. In the present study, no relationship was found between the cranial base angle and the skeletal type as determined by the ANB measurement [1]. Likewise, no differences were found between the cranial base angle and the type of mandibular rotation. The results of the present study are aligned with the findings reported by Varlela, Dhopatkar and Wilhelm, who concludes that the cranial base angle grows similarly among skeletal types I and II, without becoming more obtuse in the latter type [19–21]. An obtuse cranial base angle causes a downward and backward mandibular rotation, which would favor a type II skeletal relationship. In the present study, no relationship was found between the cranial base angle and the rotation pattern of the subjects (Table 5).

The present study included patients aged 8–12, whereupon the spheno-occipital synchondrosis was fully grown and the cranial base was therefore considered stable. However, Bjork [12] claims the growth of this structure can go up to the age of 10, from where it increases between 4 and 5 mm due to anterior apposition between 12 and 20 years old.

The cranial base angle remained relatively stable between the ages of 8 and 12. The variations found (Table 2) may be due to the cross-section nature of the study. In order to analyze changes in the type of structure, it would be necessary to carry out a longitudinal study. The stability reported in this study is aligned with the findings by Anderson [13], which show that the angle alteration occurs from birth to the age of 5. From this moment to the age of 15, it remains stable. Therefore, this would demonstrate that the structure observed in the participants of this study can be used as a reference in cephalic serial radiographs.

## Conclusion

The cranial base length influences the measurement of the angles that use it as a reference. Also, since this structure can vary among races, the mean values used must be based on measurements taken in each population.

## Abbreviations

S-N: sella–nasion
S-N-Ba: sella–nasion–basion
S-N-Ar: sella–nasion–articular
SNA: sella–nasion–A point
SNB: sella–nasion–B point
Ar-Go-Me: articular–gonion–menton
Angle ANB: angle formed between point A–point nasion and point B
